# In Memory of Professor James E. Garretson

**Published:** 1896-02

**Authors:** 


### IN MEMORY OF PROFESSOR JAMES E. GARRETSON.

A MEETING of the former students and friends of Professor James E. Garretson was held in the office of Dr. George Shidle, in Pittsburg, Pa., on November 7, 1895, Dr. A. Reinhart presiding. Drs. W. H. Fundenberg, Geo. Shidle, and Jos. Greenawalt, Committee on Resolutions, presented the following, which was adopted:

WHEREAS, In the dispensation of a wise and overruling Providence, the subject of this tribute has passed to a higher life, full of years and honors, in the zenith of his glory, and before his intellect was dimmed, or his body bowed with age.

Few men, if any, in the profession have attracted so large a share of attention, or maintained for so long a period such a degree of success.

He was indeed one of those remarkable men who stand prominently in the history of our profession as unequalled in his power of diagnosis and skill as an operator.

He possessed a wonderful faculty of communicating knowledge to his pupils, of making the most complicated subject so plain and interesting that it was rare indeed when one failed to comprehend.

To the poor and the friendless, the sick and the suffering, from the beginning to the end of his long career, he was found continually ministering.

In many respects his life had been an ideal one, a life which made the best of all that was granted it, and through it we can trace the hand of a higher one.

Sorrow so profound and general as that occasioned by the death of one so exceptionally endowed with the rarest qualities of mind, heart, and person would seem to call for general expression from those who enjoyed his friendship and benefited by his experience and writings; therefore be it

*Resolved*, That we are grateful for the privilege we enjoyed for many years in his wise and gracious leadership, and that we recall with admiration the enthusiasm, the courage, patience, and clear comprehension with which he guided us.

*Resolved*, That we will cherish his memory and endeavor to perpetuate the noble character.

*Resolved*, That a copy of this tribute be properly engrossed and sent to the family of the deceased.

*Resolved*, That a copy be sent to the dental journals for publication.

